# Hepatoprotective effects of chamazulene against alcohol-induced liver damage by alleviation of oxidative stress in rat models

**DOI:** 10.1515/biol-2020-0026

**Published:** 2020-04-20

**Authors:** Xu Wang, Ke Dong, Yujing Ma, Qizhi Jin, Shujun Yin, Shan Wang

**Affiliations:** No. 2 Ward of Hepatobiliary Surgery, Sichuan Academy of Medical Sciences, Sichuan Provincial People’s Hospital, Chengdu, Sichuan, 610047, China; Department of Echocardiography & Noninvasive Cardiology Laboratory, Sichuan Academy of Medical Sciences, Sichuan Provincial People’s Hospital, Chengdu, Sichuan, 610047, China

**Keywords:** chamazulene, oxidative stress, antioxidant enzymes, alcoholic liver injury, histopathology

## Abstract

Liver injury and disease caused by alcohol is a common complication to human health worldwide. Chamazulene is a natural proazulene with antioxidant and anti-inflammatory properties. This study aims to investigate the hepatoprotective effects of chamazulene against ethanol-induced liver injury in rat models. Adult Wistar rats were orally treated with 50% v/v ethanol (8–12 mL/kg body weight [b.w.]) for 6 weeks to induce alcoholic liver injury. Chamazulene was administered orally to rats 1 h prior to ethanol administration at the doses of 25 and 50 mg/kg b.w. for 6 weeks. Silymarin, a commercial drug for hepatoprotection, was orally administered (50 mg/kg b.w.) for the positive control group. Chamazulene significantly reduced (*p* < 0.05) the levels of serum alkaline phosphatase, aspartate aminotransferase, alanine aminotransferase, and malondialdehyde, whereas the levels of antioxidant enzymes (glutathione peroxidase, catalase, and superoxide dismutase) and reduced glutathione were significantly restored (*p* < 0.05) in contrast to the ethanol model group. The levels of pro-inflammatory cytokines (tumour necrosis factor-α and interleukin-6) were suppressed by chamazulene (*p* < 0.05) with relevance to ethanol-induced liver injury. Histopathological alterations were convincing in the chamazulene-treated groups, which showed protective effects against alcoholic liver injury. Chamazulene has a significant hepatoprotective effect against ethanol-induced liver injury through alleviation of oxidative stress and prevention of inflammation.

## 1 Introduction

Liver-related ailments are commonly associated with several causes such as genetic inheritance, toxic ingestion, viral infection, and excessive alcohol abuse. Among the causes, alcohol has a worldwide signature for liver disease [1]. The liver functions to biotransform alcohol through metabolism with internal enzymes and detoxifies by expelling alcohol out of the body. Excessive alcohol consumption leads to severe pathological changes in the liver, hence disrupting its function [2]. Alcoholic liver disease is characterized with hepatic steatosis, cirrhosis, steatohepatitis, and fibrosis. Untreated alcoholic liver disease turns into hepatocellular carcinoma, often causing mortality [3]. Although the underlying mechanism of alcoholic liver injury is not clear, oxidative stress and inflammatory response are the primary factors involved in the progression of alcoholic liver disease. Previous studies using animal models have reported increase in reactive oxygen species (ROS) and inflammatory response due to alcohol [4,5]. The activities of antioxidant enzymes and pro-inflammatory cytokines are altered due to alcoholic liver disease causing oxidative stress [6]. The activities of liver antioxidant enzymes responsible for detoxification of alcohol are reduced; therefore, the biotransformed alcohol in the form of free radicals causes damage to the hepatic cells.

Antioxidants were reported to ameliorate the effect of oxidative stress and inflammation in liver-related ailments [7,8]. Natural products are rich in bioactive compounds that are widely known to have pharmacological uses. Chamazulene is a natural proazulene majorly present in chamomile flower (*Matricaria recutita* L.). Chamomile flowers are best known to possess antioxidant, anti-inflammatory, and anti-cancer properties [9,10]. Chamazulene is a strong aromatic bioactive compound that is naturally found in essential oil extracts of several medicinal plants [11]. Pharmacological properties of chamazulene include anti-inflammatory, free radical scavenging, inhibition of cyclooxygenase-2 enzyme, and prevention of lipid peroxidation [12,13,14,15]. There are no previous studies on the hepatoprotective effects of chamazulene on animal models; therefore, this study is the first to report the liver protective effects of chamazulene against alcoholic liver injury in rat models.

## 2 Materials and methods

### 2.1 Chemicals and reagents

Chamazulene (analytical standard), ethanol (purity ≥99.8%), biochemical analysis kits, ELISA assay kit, haematoxylin and eosin (H&E), and all other chemicals and reagents were either purchased from Sigma-Aldrich (St Louis, MO, USA) or Pure One Biotechnology Ltd (Shanghai, China).

### 2.2 Experimental animals

Thirty male Wistar rats weighing 150–180 g (6–8 weeks old) were randomly divided into five groups with six rats (*n* = 6) per group. The rats were acclimatized for 1 week before the initiation of experiment, placed in plastic cages at room temperature with fresh air ventilation, and provided free access to water and chow ad libitum. The experiment was started at the beginning of June 2018 in the medical laboratory of Sichuan Academy of Medical Sciences.


**Ethical approval:** The research related to animal use complied with all the relevant national regulations and institutional policies for the care and use of animals. The animal ethical committee of Sichuan Provincial People’s Hospital gave approval for the animal experiment (ethical number: scrmyy2019072301).

### 2.3 Treatment protocol

Rats were divided into control group, ethanol-administered alcoholic model group (EtOH), low dose and high dose of chamazulene plus ethanol-treated groups, and silymarin plus ethanol-treated positive control group. Dosages for chamazulene and silymarin were chosen from preliminary tests on antioxidant activities, and duration of treatment and protocols were followed according to the previous study on alcoholic liver disease models by Ge et al. [5]. The control group received oral administration of saline in distilled water throughout the experiment. All other groups received oral administration of ethanol (50% v/v) for 6 weeks at the dose of 8 mL/kg body weight (b.w.) per day for the first 2 weeks, followed by 12 mL/kg b.w. per day for the remaining 4 weeks. Ethanol dosage for liver injury was chosen based on a similar study performed by Ge et al. [5] after confirmation through preliminary tests. Chamazulene was orally administered 1 h before the administration of ethanol at 25 mg/kg b.w. per day for the low-dose group and 50 mg/kg b.w. per day for the high-dose group for the whole 6 weeks. The positive control group rats were orally treated with silymarin at 50 mg/kg b.w. per day followed by ethanol following a similar procedure for 6 weeks. On the 43rd day after 24 h of last ethanol administration, the rats were anaesthetized to collect blood samples for serum biochemical analysis and the liver tissues were dissected for biochemical and histopathological analyses. The excised wet liver was weighed for liver index measurement. Liver index (%) was calculated by the formula: (liver weight [g]/body weight of rat [g]) × 100%. Blood samples were left to clot at room temperature and centrifuged at 3,000 rpm for 10 min to obtain serum for enzymatic (aspartate aminotransferase [AST], alanine aminotransferase [ALT], and alkaline phosphatase [ALP]) and cytokine (tumour necrosis factor-α [TNF-α] and interleukin-6 [IL-6]) analyses. Excised liver tissues were cleaned and homogenized (10%) in freshly prepared sodium phosphate-buffered saline for liver enzymatic and non-enzymatic antioxidant analyses. Portions of liver tissues were fixed in 10% formaldehyde buffer for histopathological studies. The remaining samples were frozen and stored at −80°C.

### 2.4 Determination of lipid peroxidation, reduced glutathione (GSH), and antioxidant enzymes in tissue

Biochemical analysis of tissue enzymes, GSH, and lipid peroxidation levels was performed according to the method of Li et al. [16]. Tissue homogenates (10%) were centrifuged at 3,000 rpm for 10 min at 4°C to obtain the supernatant for the determination of lipid peroxidation, GSH, glutathione peroxidase (GPx), catalase (CAT), and superoxide dismutase (SOD). Lipid peroxidation levels were analyzed using a commercial kit based on the formation of thiobarbituric acid reactive substances in conjugation with malondialdehyde (MDA) following the method of Ayidin et al. [17]. The GSH levels were assayed using a commercial kit based on the formation of 5-thiol-2-nitrobenzoic acid. The activities of antioxidant enzymes (SOD, CAT, and GPx) were determined using commercially available assay kits following the instructions provided by the manufacturer (Pure One Biotechnology Ltd) according to the method of Bacanli et al. [18]. The biochemical results were spectrophotometrically analyzed using an UV-Vis digital spectrophotometer (Shimadzu UV-1900, Japan). Total protein concentration in the liver tissues was measured by a standard Bradford protein assay.

### 2.5 Determination of serum AST, ALT, and ALP

The activities of serum biomarker enzymes for liver damage such as ALP, AST, and ALT were analyzed using commercial kits according to the instructions given by the manufacturer, following the guidelines of Gnanaraj et al. [3] and Li et al. [16]. The results for serum biomarker enzymes were given as units per litre of serum.

### 2.6 Determination of pro-inflammatory cytokines using the ELISA assay

The activities of serum pro-inflammatory cytokines (TNF-α and IL-6) were determined using ELISA assay kits (Pure One Biotechnology Ltd) according to the instructions given in the reagent kits with reference to the protocols of Xu et al. [6]. The ELISA assay results for the activities of pro-inflammatory cytokines were expressed as picograms per millilitre of serum.

### 2.7 Evaluation of liver histopathological changes

Liver tissues fixed in 10% formaldehyde buffer were dehydrated and fixed in paraffin wax, sliced to 5 µm thick films, and stained with H&E. The sections of stained liver tissues were observed under a light microscope for the evaluation of fatty changes, lymphocytic infiltration, ballooning degeneration, and hepatocellular necrosis following the method of Ge et al. [5].

### 2.8 Statistical analysis

All the values are expressed as mean ± standard error of mean. Statistical package for social sciences software (SPSS 17.0, USA) was used for statistical significance testing. Analysis of variance was performed together with Dunnett’s multiple comparison test for analysing the difference between groups. Statistical significance was considered valid for *p* values less than 0.05.

## 3 Results

### 3.1 Effects of chamazulene on the liver index of ethanol-induced liver injury in rats

The final body weight and liver index of alcoholic liver injury model rats are given in Table 1. The liver index of the ethanol-induced liver injury model group shows a significantly increased value compared to that of the normal control group. Chamazulene significantly reduced the liver index in a dose-dependent manner as compared to the ethanol control group (*p* < 0.05). Silymarin also showed a significantly lower liver index as compared to the alcoholic liver model group (*p* < 0.05).

**Table 1:** Effect of chamazulene on body weight and liver index of alcoholic liver injury in rats

Groups	Final body weight (g)	Liver index (%)
Control	220.33 ± 13.22	3.22 ± 0.46
Ethanol control	214.15 ± 12.18	4.88 ± 0.50^a^
Chamazulene 25 mg/kg b.w. + EtOH	216.64 ± 11.68	4.26 ± 0.38^b^
Chamazulene 50 mg/kg b.w. + EtOH	215.55 ± 10.14	3.85 ± 0.43^b^
Silymarin 50 mg/kg b.w. + EtOH	217.20 ± 11.26	3.42 ± 0.34^b^

Values are expressed as mean ± standard error of mean (*n* = 8) and analyzed by one-way analysis of variance followed by Dunnett’s multiple comparisons test. The symbol ‘a’ represents significance (*p* < 0.05) as compared to the normal control group, whereas the symbol ‘b’ represents significance (*p* < 0.05) as compared to the ethanol control group.

### 3.2 Chamazulene restores GSH and antioxidant enzyme activities in alcoholic liver injury

**Figure 1:**
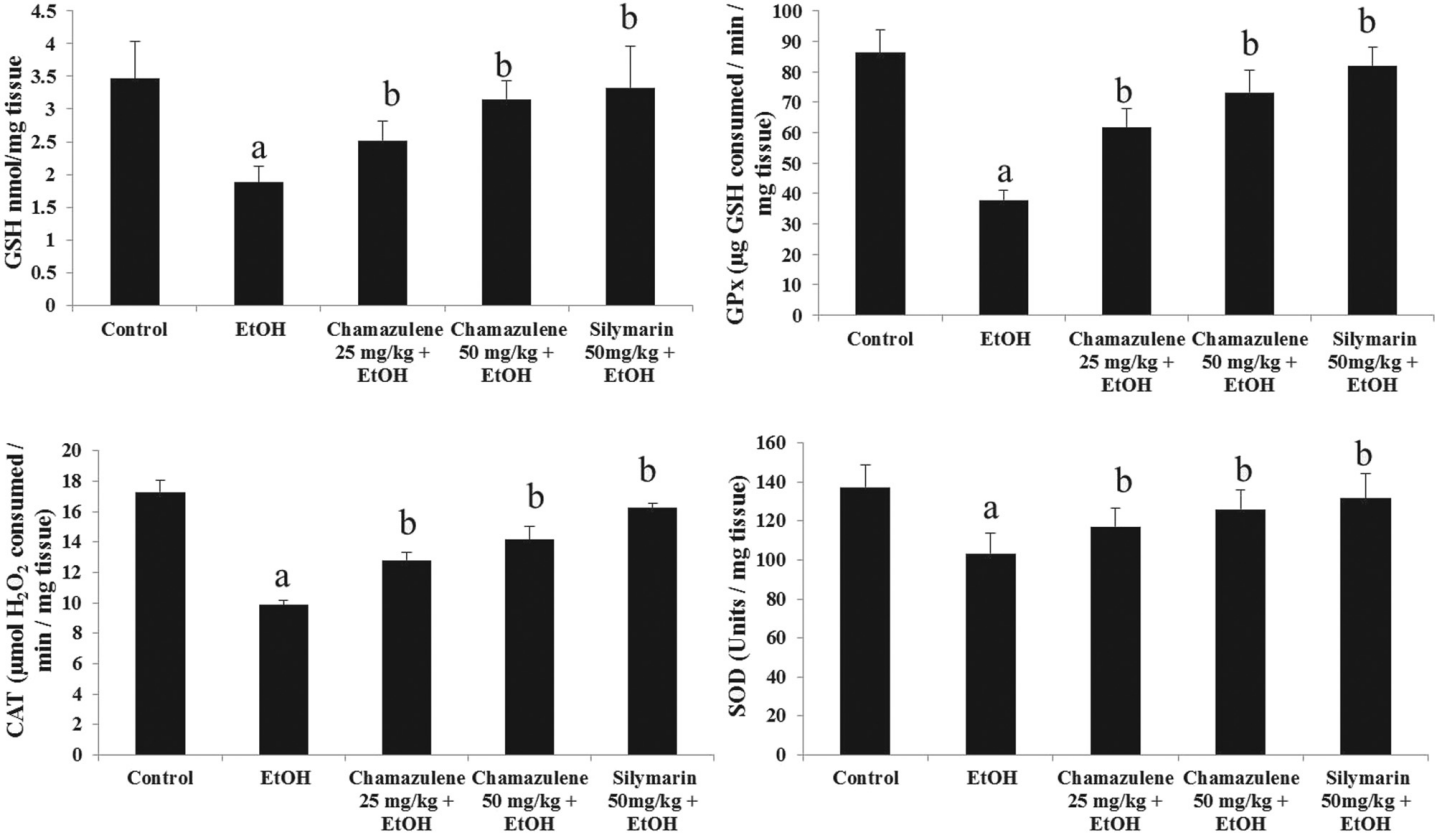
Effect of chamazulene on the activities of non-antioxidant GSH and antioxidant enzymes (GPx, CAT, and SOD) against ethanol-induced liver injury. Results are displayed as the mean analysis of six rats (*n* = 6) and standard error of mean. The symbol ‘a’ represents significance (*p* < 0.05) as compared to the normal control group, whereas the symbol ‘b’ represents significance (*p* < 0.05) as compared to the ethanol control group. GSH = reduced glutathione; GPx = glutathione peroxidase; CAT = catalase; SOD = superoxide dismutase.

The levels of GSH and activities of antioxidant enzymes (SOD, GPx, and CAT) in ethanol-induced liver injury are shown in Figure 1. It is clearly shown that GSH was largely consumed in the ethanol-induced model group as compared to the normal control group (*p* < 0.05). Chamazulene significantly restored the GSH levels comparably better than the ethanol-induced model group (*p* < 0.05). Silymarin also showed significantly increased levels of GSH as compared to those of the ethanol-induced model group (*p* < 0.05). The activities of antioxidant enzymes, SOD, CAT, and GPx, were drastically depleted in the ethanol-induced model group (*p* < 0.05), but chamazulene increased the activities of antioxidant enzymes at both 25 mg/kg b.w. and 50 mg/kg b.w. doses (*p* < 0.05). The results were comparable to positive control silymarin.

### 3.3 Chamazulene prevents MDA formation and decreases serum ALP, AST, and ALT levels

**Figure 2:**
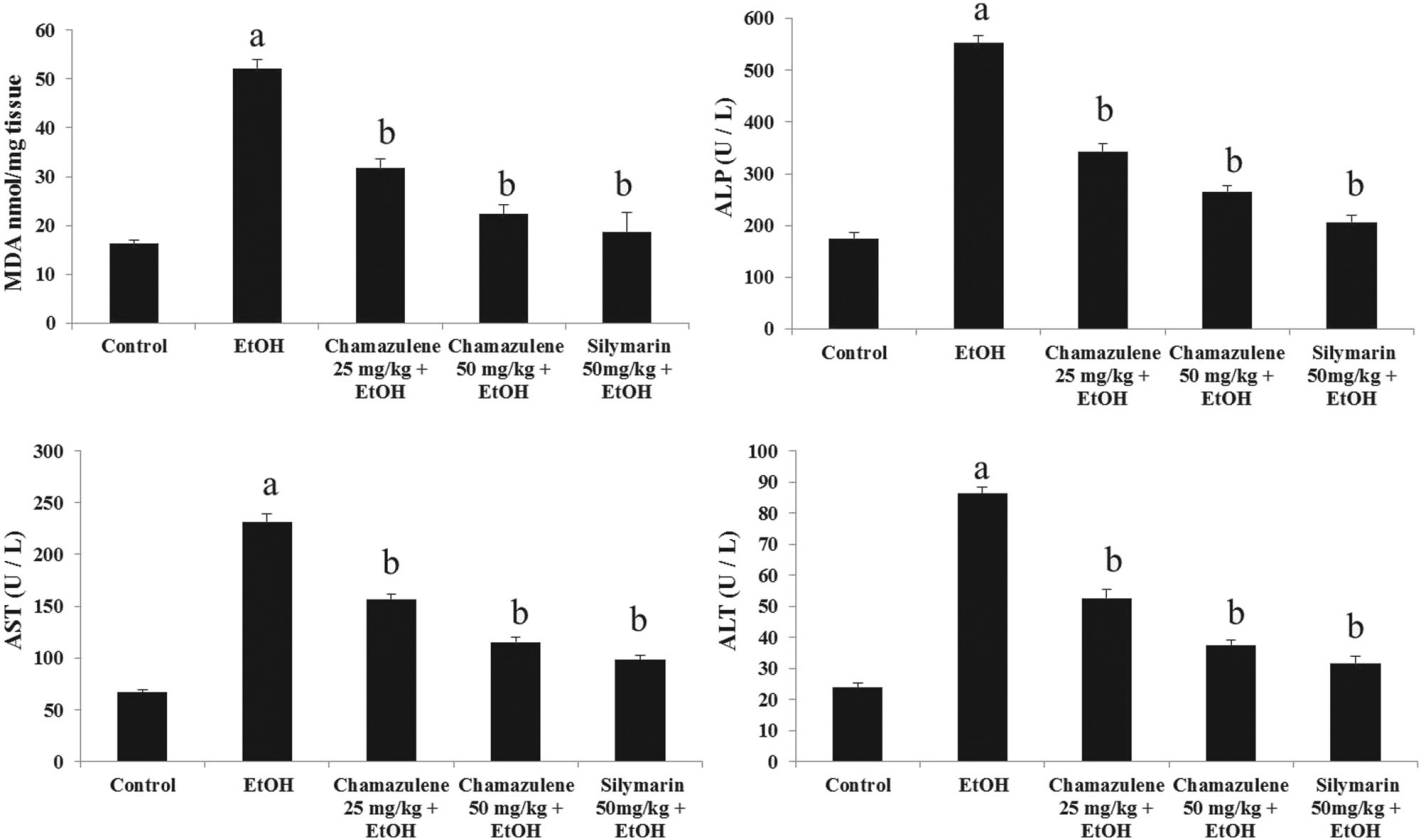
Effect of chamazulene on the levels of MDA formation and serum liver injury marker enzymes (ALP, AST, and ALT) against ethanol-induced liver damage. Results are displayed as the mean analysis of six rats (*n* = 6) and standard error of mean. The symbol ‘a’ represents significance (*p* < 0.05) as compared to the normal control group, whereas the symbol ‘b’ represents significance (*p* < 0.05) as compared to the ethanol control group. MDA = malondialdehyde; ALP = alkaline phosphatase; AST = aspartate aminotransferase; ALT = alanine aminotransferase.

The effects of chamazulene on MDA formation and serum biomarker enzymes for liver injury such as ALP, AST, and ALT are shown in Figure 2. The MDA level was clearly high in the ethanol-induced model group compared to that of the normal control group (*p* < 0.05). Chamazulene at both 25 mg/kg b.w. and 50 mg/kg b.w. doses significantly prevented MDA formation in comparison to the ethanol-induced model group (*p* < 0.05). The activities of serum biomarker enzymes (AST, ALT, and ALP) were significantly high in the ethanol-induced model group compared to those of the normal control group (*p* < 0.05). Chamazulene dose dependently was able to suppress the levels of serum AST, ALT, and ALP compared to the ethanol-induced model group (*p* < 0.05). Silymarin significantly reduced the levels of MDA, ALP, AST, and ALP in contrast to the ethanol-induced model group (*p* < 0.05).

### 3.4 Preventive effects of chamazulene on the levels of pro-inflammatory cytokines

**Figure 3:**
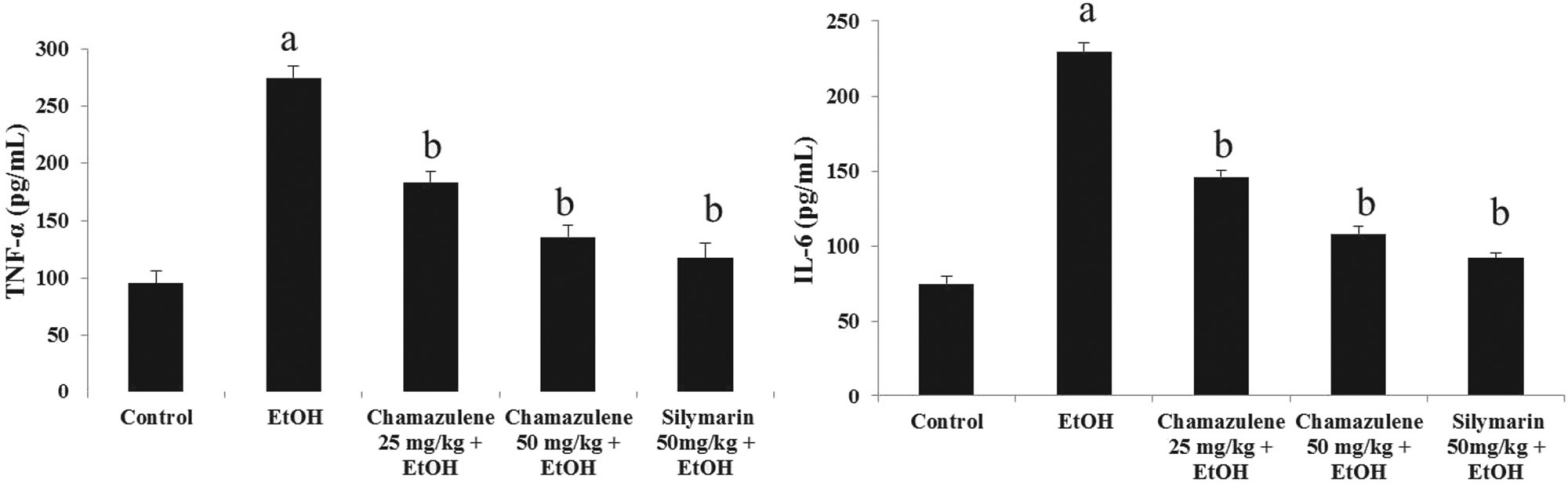
Effect of chamazulene on pro-inflammatory cytokines (TNF-α and IL-6) against ethanol-induced liver damage. Results are displayed as the mean analysis of six rats (*n* = 6) and standard error of mean. The symbol ‘a’ represents significance (*p* < 0.05) as compared to the normal control group, whereas the symbol ‘b’ represents significance (*p* < 0.05) as compared to the ethanol control group. TNF-α = tumour necrosis factor-α; IL-6 = interleukin-6.

ELISA assay results for the levels of pro-inflammatory cytokines (TNF-α and IL-6) in ethanol-induced liver injury are shown in Figure 3. The levels of TNF-α and IL-6 were significantly elevated in the serum of the ethanol-induced liver damage group as compared to those of the normal control group (*p* < 0.05). Chamazulene was able to suppress the levels of TNF-α and IL-6 in the serum of the 25 mg/kg b.w. treated group and 50 mg/kg b.w. treated group in contrast to the ethanol-induced model group (*p* < 0.05). Silymarin also suppressed the serum levels of TNF-α and IL-6 in comparison to the ethanol-induced model group (*p* < 0.05).

### 3.5 Histopathological changes in alcoholic liver injury due to chamazulene administration

**Figure 4:**
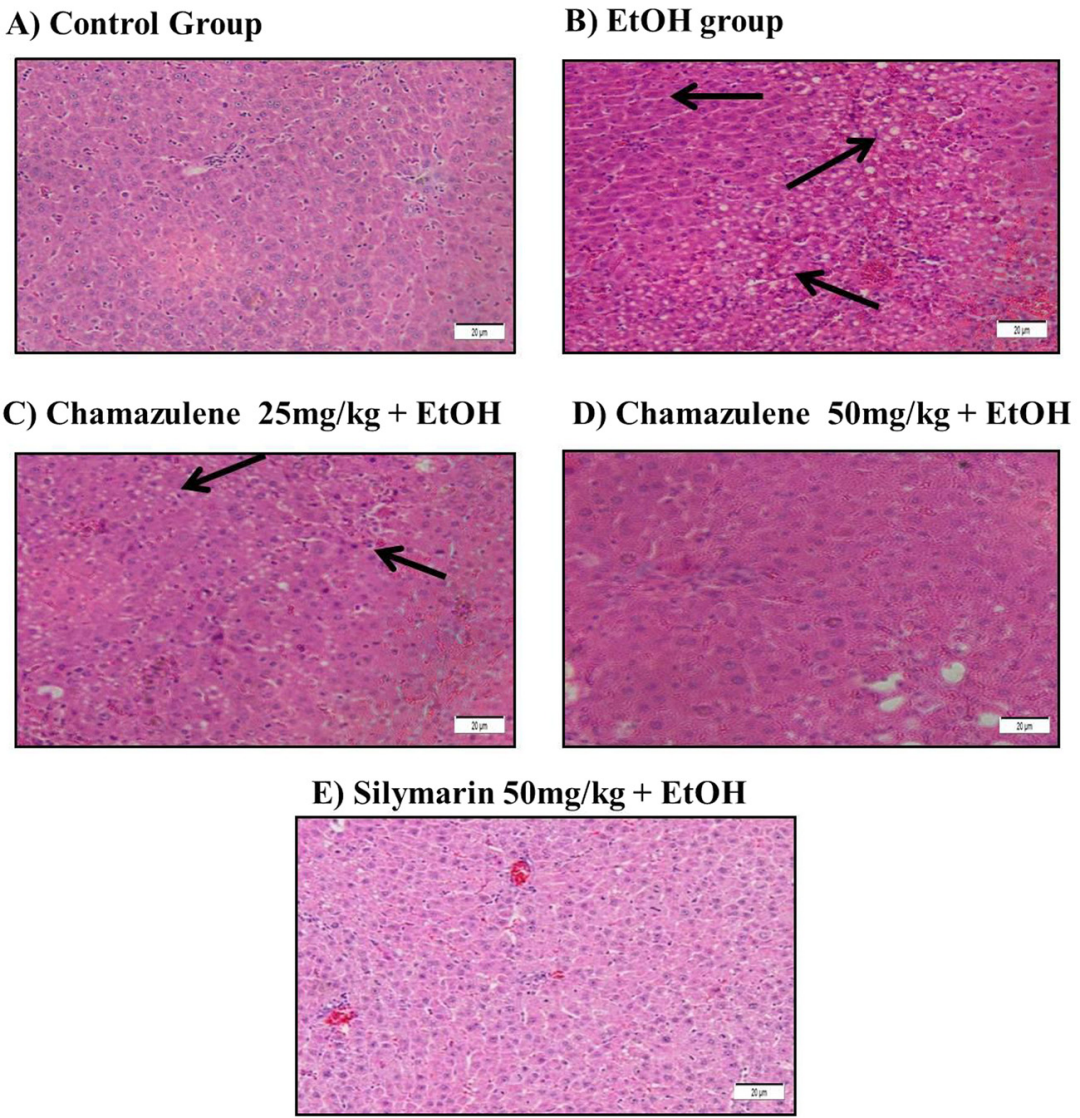
H&E staining of ethanol-induced liver damage and the protective effects of chamazulene at 100× magnification. (A) Control group: normal arrangement of hepatocytes and sinusoidal spaces; (B) alcohol control group: heavy loss of sinusoidal spaces, presence of necrotic cells, lymphocytic infiltration, ballooning degeneration, and fatty changes are observed, indicated by black arrows; (C) chamazulene (25 mg/kg b.w. + EtOH) group: there are several necrotic hepatocytes and lymphocytic infiltrations present, indicated by black arrows but mostly sinusoidal spaces show recovery; (D) chamazulene (50 mg/kg b.w. + EtOH) group: hepatocytes show signs of protection against ethanol-induced liver injury with distinct sinusoidal spaces; and (E) positive control group: silymarin as a positive control significantly protected the liver against ethanol-induced injury which shows arrangement of hepatocytes almost similar to normal liver.

Histopathological alterations (H&E) in the alcoholic liver injury model and protective effect of chamazulene are shown in Figure 4. The morphological arrangement of liver cells in the normal control group showed healthy signs with regular hepatocyte arrangements, distinct nucleus, and sinusoids. In contrast, the hepatocytes of the ethanol-induced model group showed extensive derangement with loss of sinusoidal spaces, inflammatory signs with cell infiltrations were observed, necrotic cells were found with loss of nuclear integrity, ballooning degeneration, and fatty changes were clearly visible. Chamazulene administration reversed the morphological changes caused by ethanol-induced liver damage by restoring the hepatocyte arrangements, reduced inflammatory cell infiltration, and preserved sinusoidal spaces. The silymarin-treated group showed positive morphology of liver with arrangement of hepatocytes almost similar to normal liver.

## 4 Discussion

Excessive alcohol consumption is becoming a trend among younger generations worldwide [2]. Therefore, most people are susceptible to alcoholic liver damage. This study emphasized the importance of antioxidant compounds in reversing the effect of alcoholic liver injury. Chamazulene, a bioactive compound with antioxidative properties, was studied to determine its ability to prevent alcoholic liver damage in ethanol-induced liver injury of rat models. Oxidative stress has a major role in the pathogenesis of alcoholic liver injury through breakdown of the cell membrane by oxidation. Alcohol intake boosts the ROS generation within the liver, thus defeating the antioxidant defence mechanism that is supposed to scavenge the ROS [4]. It was found that chamazulene significantly prevented oxidative stress by alleviating the activities of antioxidant enzymes and GSH levels towards normal. The ethanol-induced liver injury model group expressed high state of oxidative stress where the levels of GSH and activities of antioxidant enzymes (SOD, GPx, and CAT) were depleted. Similarly, previous studies by Zhang et al. [19] and Zhu et al. [20] also reported the revival of antioxidant defence upon antioxidant administration. Lipid peroxidation has been reported to be the most dangerous reaction in ethanol-induced liver damage [8]. Excessive alcohol consumption leads to overproduction of MDA in liver due to lipid peroxidation, causing hepatic injury and apoptosis [6]. Pre-treatment with chamazulene was able to prevent free radicals from oxidizing the hepatocellular membranes. This was proven from the results of lipid peroxidation where the MDA levels in the ethanol-induced liver injury model group were elevated above normal but chamazulene treatment significantly reduced the MDA levels towards normal. The silymarin-treated positive control group also exhibited reduced levels of MDA formations. It is well observed that natural antioxidants are able to prevent oxidative stress by donating electrons to scavenge free radicals. Similar results were presented in previous studies in support of the results of reduced MDA levels by antioxidant compounds in alcoholic liver injury models [19,20].

Enhanced activities of serum enzyme markers (AST, ALT, and ALP) are commonly known as liver damage indicators [4]. Serum biomarker enzymes (ALP, AST, and ALT) were largely increased in the ethanol-induced liver injury model group, indicating that the liver damage caused the leakage of these enzymes located within the hepatocytes into blood stream. The chamazulene-treated groups showed significantly reduced levels of serum ALP, AST, and ALT towards normal, which proves that the compound well protected the structure of hepatocytes. These results can be attributed to the prevention of lipid peroxidation by chamazulene as discussed earlier. These findings are in agreement with the reports of Ge et al. [5] and Taner et al. [21], indicating the prevention of serum biomarkers by antioxidant compounds in the ethanol-induced liver injury model. Besides oxidative stress, inflammation is also a common occurrence in alcoholic liver disease. Alcohol triggers the release of pro-inflammatory cytokines leading to aggravation of inflammatory response causing the development of liver damage [4]. TNF-α and IL-6 are the most persistent pro-inflammatory cytokines featured in ethanol-induced liver injury models [22]. TNF-α and IL-6 in the serum determined by the ELISA assay showed highly increased activity in the ethanol-induced liver injury model group, due to the inflammatory reaction caused by liver injury. The inflammatory response was significantly suppressed by chamazulene by reducing the activities of pro-inflammatory cytokines (TNF-α and IL-6). Antioxidant compounds are known to possess anti-inflammatory activity through suppression of pro-inflammatory cytokines [23]. Suppression of TNF-α and IL-6 by chamazulene could be credited to its antioxidative and anti-inflammatory abilities. Anti-inflammatory potential of chamazulene in the alcoholic liver damage model is in agreement when compared with previous findings of Flemming et al. [10] and recent research studies on antioxidant compounds in alcoholic liver injury models [6,16,19].

Alcoholic liver damage is normally characterized with changes in the hepatocellular morphology [20]. Histopathological alterations in the ethanol-induced liver injury model group evidenced the extent of liver injury, relating back to the biochemical results of lipid peroxidation, serum biomarker enzymes, and pro-inflammatory cytokines. Administration of chamazulene widely prevented liver injury through preservation of hepatocellular membrane integrity, prevention of oxidative stress, and suppression of inflammatory responses. The histopathological findings are comparable with the results of Xu et al. [6] and Al-Attar and Alomar [24], demonstrating the levels of hepatocyte damage. Collectively, this study proves that chamazulene has remarkable hepatoprotective effects against alcoholic liver injury through alleviation of oxidative stress and suppression of pro-inflammatory cytokines.

## 5 Conclusion

In this study, chamazulene has shown its ability as a protective compound against oxidative stress and inflammatory response in the alcoholic liver injury model. Chamazulene significantly reversed the effect of ethanol-induced liver injury through modulation of liver marker enzymes, antioxidant enzyme activities, pro-inflammatory cytokines, and MDA formations. The extent of liver protection shown by chamazulene against ethanol-induced liver damage in rats proves that chamazulene could be commercialized as a pharmaceutical drug for liver ailments. Further research is needed to validate the antioxidant effect of chamazulene on other diseases caused by manifestation of oxidative stress and inflammation.
